# Photonic–Plasmonic Nanostructures for Solar Energy Utilization and Emerging Biosensors

**DOI:** 10.3390/nano10112248

**Published:** 2020-11-12

**Authors:** Van Tan Tran, Huu-Quang Nguyen, Young-Mi Kim, Gyeongsik Ok, Jaebeom Lee

**Affiliations:** 1 Department of Chemistry, Research Institute of Materials Science, Chungnam National University, Daejeon 34134, Korea; trantan160288@gmail.com (V.T.T.); nghquang812@gmail.com (H.-Q.N.); 2 Faculty of Biotechnology, Chemistry and Environmental Engineering, Phenikaa University, Hanoi 12116, Vietnam; 3 Department of Chemical Engineering and Applied Chemistry, Chungnam National University, Daejeon 34134, Korea; dtp04017@naver.com; 4 Research Group of Consumer Safety, Korea Food Research Institute (KFRI), Wanju 55365, Korea; gsok@kfri.re.kr

**Keywords:** photonic–plasmonic, nanostructure, solar energy, light harvesting, biosensor, terahertz

## Abstract

Issues related to global energy and environment as well as health crisis are currently some of the greatest challenges faced by humanity, which compel us to develop new pollution-free and sustainable energy sources, as well as next-generation biodiagnostic solutions. Optical functional nanostructures that manipulate and confine light on a nanometer scale have recently emerged as leading candidates for a wide range of applications in solar energy conversion and biosensing. In this review, recent research progress in the development of photonic and plasmonic nanostructures for various applications in solar energy conversion, such as photovoltaics, photothermal conversion, and photocatalysis, is highlighted. Furthermore, the combination of photonic and plasmonic nanostructures for developing high-efficiency solar energy conversion systems is explored and discussed. We also discuss recent applications of photonic–plasmonic-based biosensors in the rapid management of infectious diseases at point-of-care as well as terahertz biosensing and imaging for improving global health. Finally, we discuss the current challenges and future prospects associated with the existing solar energy conversion and biosensing systems.

## 1. Introduction

The application of solar energy in addressing global energy and environmental issues has recently attracted increasing attention, and various techniques for the same have emerged. Solar energy can be converted directly into electrical or chemical energy through photovoltaics or photoelectrochemical cells and photocatalysis, respectively, which is generally governed by four processes: light absorption, charge separation, charge migration, and charge recombination. However, as individual semiconductor materials cannot be optimized for all four processes, the overall efficiency of solar conversion is very low, hindering their practical applications. There are two main reasons for the low efficiency of solar energy conversion: limited light absorption and high recombination rates of charge carriers. The first reason is large bandgaps of the most commonly used photocatalysts such as TiO_2_ and ZnO, which is the main challenge in their solar energy conversion applications. The second reason for the low efficiency of solar energy conversion is the high electron–hole recombination rate in the bulk of a semiconductor owing to the short diffusion length of photoexcited minority charge carriers.

Several approaches have been proposed to overcome the limitations of the existing semiconductors. For example, ultraviolet (UV) bandgap semiconductors were doped to increase the absorption of visible light. However, this approach leads to high charge recombination rates due to the detrimental charge mobility caused by isolated midgap states [1]. Sensitizers are also used to convert absorbed visible-light radiation to electricity and transfer the photoexcited charge carriers to semiconductor support. Organic dyes or quantum dots are generally used in sensitizers; however, both are plagued by photostability issues, particularly when used in photocatalysis or photoelectrochemical cells. Integrating photonic or plasmonic nanostructures with semiconductors has been proven as an alternative to improve the solar energy conversion efficiency [2]. The resonant scattering near photonic bandgap and slow-photon effects of photonic crystals (PCs) can effectively improve interactions between semiconductors and light. The enhancement of light absorption in the semiconductor induced by plasmonic nanostructures is attributed to their ability to increase the path length of light and concentrating the incident electromagnetic field. Further, the energy transformed from the metal to the semiconductor can induce charger separation, reducing the electron–hole recombination rate in the semiconductor.

The current health crisis caused by infectious diseases also compels us to develop next-generation diagnostics. Photonic- and plasmonic-based platforms have been proven to be the key candidates for biomolecular research and biomedical applications. Owing to their unique structural and optical features, PC-based nanostructures have been proven as an important diagnostic tool to effectively control infectious diseases. Biosensing using PCs is mostly based on changes in the refractive index due to the molecules or pathogens that are in the vicinity of the PCs. Furthermore, plasmonic nanostructures, which can generate intense and highly localized electromagnetic (EM) fields, have been demonstrated to be extremely sensitive transducers to track small local refractive index changes caused by the adsorption of biomolecules to their surface [3]. A variety of diagnostic methods have emerged based on photonic, plasmonic nanostructures, including colorimetric assays, surface-enhanced Raman spectroscopy (SERS), surface plasmon polariton (SPP), optical waveguide, and terahertz sensing and imaging.

Herein, the applications of photonic and plasmonic nanostructures in solar energy conversion and biosensing are reviewed. For solar energy conversion, three main applications are reviewed: photovoltaic, photothermal, and photocatalytic applications. We discuss different approaches to enhance solar energy conversion efficiency by incorporating semiconductors with photonic, plasmonic, or hybridized photonic–plasmonic nanostructures. Furthermore, the mechanism of various sensing modalities, such as photonic, plasmonic, and plasmonic–photonic colorimetric assays, and recent advancements in biosensing strategies to achieve clinically relevant performances within the POC feasible frameworks will be discussed. Finally, we provide an overview of the challenges and prospects of the current research studies on the photonic–plasmonic-based solar energy conversion and emerging biosensors.

## 2. Photovoltaics

### 2.1. Photonic Crystal-based Broadband Antireflective Surfaces

**Figure 1:**
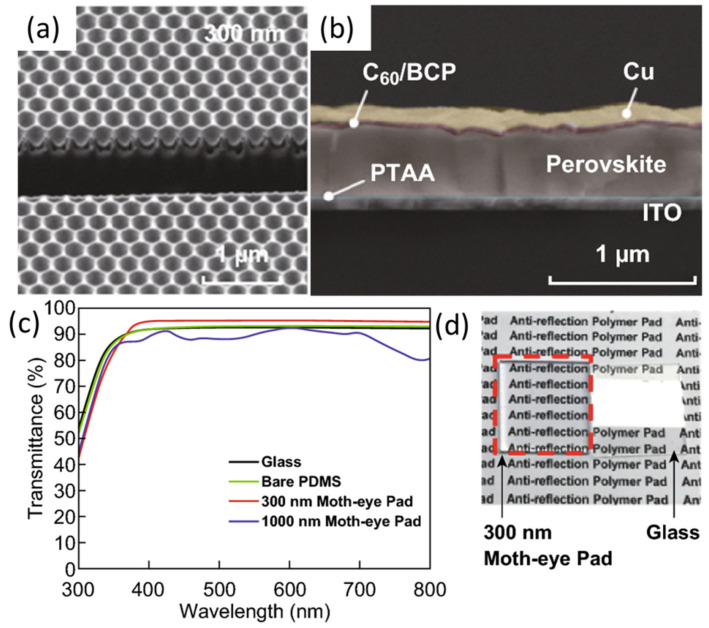
Nanostructured moth-eye antireflective surface. Effective antireflective (AR) technique for the solar spectrum. (**a**) Focused ion-beam-assisted cross-sectional scanning electron microscopic images of a moth-eye PDMS film with a 300 nm periodicity. (**b**) Perovskite solar cell (PSC) configuration imaged in a back-scattered electron mode. (**c**) Transmittance spectra of the differently prepared samples of glass, bare PDMS, and moth-eye films with 300 and 1000 nm periodicities. (**d**) Digital camera image demonstrating the antireflective effect of the 300 nm moth-eye PDMS film. Diameter and height of the hexagonal array are equal to periodicity in all PDMS films. These figures have been reproduced with permission from [12]. Copyright Springer, 2019.

Photovoltaic (PV) devices that convert solar energy into electrical power are among the most researched devices for solar energy conversion. Enhancing light absorption is one of the key factors determining the overall conversion efficiency, especially in thin-film solar cells. To improve light trapping, it is important to understand the types of nanostructures and their optimal spatial arrangement. For example, by manipulating the structure of photonic nanostructures, the light–matter interaction can be enhanced via increasing the optical path length within the absorbing medium and their interaction times, resulting in enhanced optical absorption [4,5]. The high reflection loss at the glass–air interface, which degrades the performance of solar cells, can be reduced by an antireflective (AR) coating. However, a narrow AR bandwidth and small incidence angle range of conventional AR coatings restrain the solar cell performance. An alternative approach to suppress reflection losses over a wide range of wavelengths has been widely introduced in various types of solar cells by producing a gradual reduction of refractive index away from the solar cell top surface. Based on this strategy of broadband reflection suppression, the performance of various PV devices, including perovskite and silicon solar cells [6,7], germanium-based PV cells [8], and gallium arsenide (GaAs)-based solar cells [9,10], was significantly increased. The form of moth-eye antireflective schemes, which are also referred to as nanotapered arrays, is among the most effective approaches for effective AR over a wide range of useful solar spectra. A single-junction Ge/InGaP solar cell with a PC-nanostructured surface showing antireflective properties was one of the very first experimental applications utilizing this effect [11]. Kim et al. demonstrated nanostructured PDMS films inspired by moth-eyes fabricated using a soft lithography method, showing good structural fidelity and notably improved performance of perovskite solar cells (PSCs) [12]. Highly efficient PSCs, with a power conversion efficiency exceeding 20%, were obtained with 300 nm periodic structures (Figure 1a–d). Similarly, Choi et al. reported that optimized AR surfaces are composed of moth-eye-patterned polyurethane-acrylate of 300 nm [13]. Baquedano et al. assessed the optical and surface properties of nanostructured antireflective solar glass, which saw an increase in transmission and wettability in 1D- and 2D-ordered nanostructures compared to those of disordered nanostructures [14].

### 2.2. Photonic Crystal-based Omnidirectional Antireflective Surfaces

Light trapping from large angles of incidence is another strategy to enhance the conversion efficiency of a PV cell. Luo et al. developed SiO_2_ nanosphere-based AR coatings using a facile, inexpensive, and scalable spin-coating technique. Because of the isotropic photonic structures of SiO_2_ nanospheres, the optimized coating exhibits enhanced transmittance over a broadband of 400–800nm and less angular dependence for incident light, increasing the omnidirectional PV performance of solar cells [15]. Various photonic-based AR coatings inspired by insects and plants have also been developed to improve PV performance, particularly in enhancing the light-harvesting efficiency in dye-sensitized solar cells (DSSCs) [16]. Yun et al. proposed a strategy to enhance the performance of DSSCs by designing an omnidirectional light-capturing layer that mimics structural epidermal cells in leaf structures. Light-harvesting layers with different shapes inspired by the epidermal structures of various plant species were fabricated and incorporated into DSSCs, resulting in enhanced capturing efficiency of obliquely incident light up to 70% [17].

### 2.3. Mechanisms for Incorporating Plasmonic Nanostructures in Solar Cell Systems

Incorporating plasmonic nanostructures in solar cell systems is an alternative option for improving PV conversion efficiency [18]. Besides enhancing light–matter interactions, surface-plasmon-enhanced local electric fields can also effectively suppress the electron–hole recombination in semiconductors. The mechanisms of the plasmonic-enhanced solar cell include: (1) light trapping, (2) hot-electron injection, and (3) local electromagnetic field enhancement. Here, we briefly discuss these mechanisms.

#### 2.3.1. Light Trapping

Thin-film solar cells with microscale thickness have attracted considerable interest because of their unique advantages over the thicker counterparts, including an increased open-circuit voltage (*V*_OC_) and cost/time-effective processes. However, one of the main issues of thin-film solar cells limiting their performance is the low absorption coefficient. Due to the unique scattering properties, large metallic nanoparticles (NPs) can increase the optical path length, leading to effective light trapping within the semiconductor that has a much smaller layer thickness compared to the material’s intrinsic absorption length. Positioning metallic NPs on the surface of a semiconductor can induce a forward directional scattering into the semiconductor. Light scattered at angles exceeding the critical angle of reflection can also be effectively trapped within the solar cell when equipped with a metallic reflector on its back. Therefore, the optical path length can be efficiently increased to trap incident light since it is concentrated into the semiconductor and travels multiple times through it. Using this strategy, by incorporating the solar cell with an Al NP array, the absorption rate and current density of thin-film GaAs solar cells were significantly enhanced up to 0.7983 and 25.77 mA/cm^2^, respectively [19]. Other strategies using nanostructured plasmonic thin films and metallic grating structures were proposed to effectively enhance light coupling, trapping, and absorption in ultrathin solar cells [20,21,22,23,24]. An average absorption of 90% over a broadband of 400 to 900 nm along with near independence of light polarization and an incident angle over a range of 0°–75° was achieved using a thin plasmonic cavity consisting of a 30 nm thick Au metallic mesh electrode with a subwavelength hole-array [25]. The impressive light-trapping enhancement of the plasmonic nanohole arrays was attributed to multiple cavity modes and surface plasmon modes in the structure.

#### 2.3.2. Hot-Electron Injection

**Figure 2:**
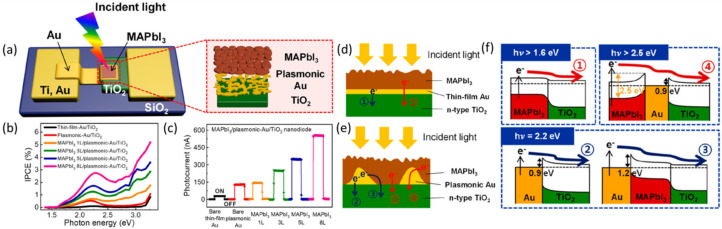
Generation of plasmonic hot electrons. (**a**) Schematic of the MAPbI_3_/plasmonic Au/TiO_2_ nanodiode (left) and magnified illustration of the active area (right). (**b**) Incident photon-to-electron conversion efficiency (IPCE) as a function of incident photon energy. (**c**) Short-circuit photocurrents measured on the MAPbI_3_/plasmonic Au/TiO_2_ nanodiodes with respect to the number of MAPbI_3_ layers. Schematic drawing of the hot-electron pathways for the MAPbI_3_/thin-film Au/TiO_2_ (**d**) and the MAPbI_3_/plasmonic Au/TiO_2_ (**e**) structure. (**f**) Theoretically drawn energy levels of the MAPbI_3_/plasmonic Au/TiO_2_ structure. Reproduced with permission from [30]. Copyright American Chemical Society, 2019.

This mechanism is based on the injection of plasmon-induced hot electrons with sufficient energy to overcome the Schottky barriers at the metal/semiconductor into the conduction band of the neighboring semiconductor [26,27]. Au nanorods (NRs) were used to generate the majority of charge carriers in a plasmonic PV device, which are energetic enough to clear the Schottky barrier or quantum-mechanically tunnel through it, thereby producing the output photocurrent [28]. Under short-circuit conditions, hot electrons with visible wavelength incident photon-to-electron conversion efficiency (IPCE) values of ~1.0% and IQE of 2.75% were extracted, which are considerably higher than those previously reported for plasmonic photovoltaics under zero-bias conditions. Using Ag nanoplates integrated between Si nanowires (NWs) and PEDOT/PSS, Lui et al. proposed a concept to absorb and convert near-infrared (NIR) light into hot electrons. The hot electrons overcame the Schottky barrier between Si and Ag and flowed into the conduction band of the Si NWs. The external quantum efficiency of flexible NIR PV devices was improved by 59% using this strategy [29]. Park et al. recently developed a strategy to enhance the lifetime and flux of plasmon-nduced hot electrons, which are decisive for developing practical hot-carrier solar cells [30]. Nanodiodes consisting of a perovskite layer CH_3_NH_3_PbI_3_ (MAPbI_3_) were stacked on a plasmonic Au/TiO_2_. They found that quantum efficiency and the short-circuit photocurrent were increased by the deposition of MAPbI_3_ on both continuous thin-film Au and randomly connected Au nanostructures. They also confirmed that integrating MAPbI_3_ with the Au nanostructure notably prolongs hot-electron lifetimes, which can be associated with the high photocurrent in nanodiode (Figure 2).

#### 2.3.3. Local EM Field Enhancement

As per this mechanism, the strong enhancement of the local EM field induced by localized plasmon resonances of the metal nanoparticles can also promote the generation of photoexcited electrons and holes in solar cells [31]. This strategy is usually implemented by embedding plasmonic nanostructures in the active layer, which act as optical antennas to store the incident light energy in the localized surface plasmon resonance (LSPR), leading to effective enhancement of the light absorption in the solar cell. For example, Bayle et al. experimentally proved plasmonic near-field enhanced light absorption by embedding Ag NPs in organic metal halide perovskite thin-films [32]. Light-harvesting at the maximum resonance frequencies and over the entire spectral range was increased up to 80% and 20%, respectively. Au NRs capped with AgS_2_ were integrated into the photoanode of DSCs to enhance longer wavelength sunlight utility, leading to an improvement in photocurrent by 37.6% in the spectral region of 600–720 nm due to the longitudinal plasmon resonance of Au NRs [33]. Gold nanorods (Au NRs) and magnesium oxide were more recently introduced into the mesoporous perovskite solar cells, which resulted in the improvement of near-field and far-field solar energy conversion of over 15% [34]. The embedded Au NRs enhanced PV performance by utilizing the longitudinal plasmon resonances (LPRs), which also support higher absorption and scattering cross-sectional efficiency, improving the EM field intensity significantly, especially at the wavelengths near the LPR peak (665 nm). In another study, Shen et al. reported plasmon-enhanced MAPbI_3_ thin-film perovskite solar cells using nanopatterned plasmonic arrays [35]. The calculated PCE of 45.5% was found in the nanopatterned plasmonic solar cell compared to its flat counterpart.

At present, the use of photonic and plasmonic nanostructures in PV devices has advantages and disadvantages. However, combining photonic crystal and plasmonic nanostructures with solar cell devices can induce synergetic light manipulating for resonant-enhanced light harvesting and power conversion. Li et al. proposed a coupling architecture of an Au NP-impregnated inverse opal TiO_2_ film to enhance the visible and NIR light absorption of DSCs [36]. The light absorption and the overall light-to-electricity conversion yield were increased up to 62% and 41%, respectively, compared with the control test.

## 3. Photothermal Conversion

The Shockley–Queisser limit, which is caused by the radiative recombination and sub-bandgap transition losses of semiconductors, defines the maximum theoretical efficiency of a single-junction PV device. Although extensive studies have been conducted to overcome practical problems with PV cells, such as reflection and surface defects, solar–thermal conversion approaches may provide an alternative method for overcoming this theoretical limit and increasing the solar energy capture and conversion efficiency. The nonradiative decay process of the surface plasmons, once thought to be only a burden limiting plasmonic device performances, has recently been proven to be significantly more useful in the form of hot electrons for solar–thermal conversion. Furthermore, photonic and nanophotonic structures could be effectively deployed as an integrated system of absorbers and thermal emitters for PV devices. These structures produce approaches to manipulate EM waves on micro- and nanoscales by modulating design parameters such as periodicity and lattice orientation. In this section, solar–thermal applications, such as solar thermophotovoltaics (STPV), solar steam generation (SG), and radiative cooling, are discussed.

### 3.1. Plasmonic Nanostructure-based Solar Thermophotovoltaics

**Figure 3:**
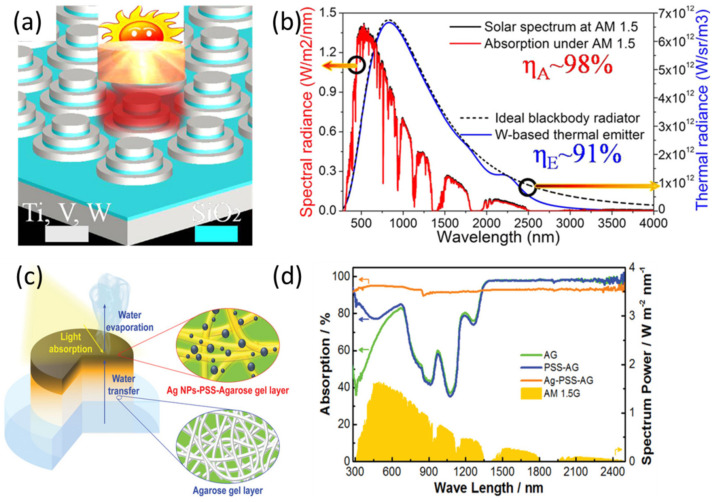
Preparation and application of thin-film solar thermophotovoltaics. (**a**) Schematic diagram of the Ti-based solar absorber. (**b**) Direct comparison between the standard spectrum of solar radiance under an air mass (AM) of 1.5 (solid line) and the solar energy absorption of the solar absorber under an AM of 1.5 (dotted line). Absorption efficiency of the solar absorber (dashed line). Reproduced with permission from [39]. Copyright Elsevier, 2019. (**c**) Schematic illustration of the double-layer solar vapor generation device. The top silver-poly (sodium-p-styrenesulfonate)-agarose gel (A-gPSS-AG) layer serves as the light-harvesting module, while the bottom AG layer acts as the water-transfer module. (**d**) UV–vis-NIR absorption spectra of AG, PSS-AG, and Ag-PSS-AG samples. Reproduced with permission from [41]. Copyright Wiley, 2019.

In an STPV system, thermal energy is generated using the absorption and conversion of solar irradiation, which is further applied to the emitter and increases the temperature of the PV cell in a controlled manner. As a result, low-energy emissions, mostly in the near-infrared and infrared regions, are emitted thermally into the cell to produce charge carriers. Since the EM absorber is the main component differentiating STPV devices from other PV systems, various techniques to enhance light absorption by a thin-film absorber have been developed and studied to improve STPV performance. In an STPV system, a selective solar irradiation absorber is used to heat a selective emitter. This combined absorber–emitter structure can be efficiently designed to absorb a broad range of solar radiation, turning this energy into narrow-band emission matching the bandgap of the PV cell. Significant advances in the research on metal–insulator–metal (MIM) plasmonic absorbers have been made over the last decade. MIM designs can facilitate efficient light trapping across the full spectrum, i.e., from UV to infrared (IR), which could be effectively used in PV applications to achieve a broad spectral range and high efficiency with less material consumption. There are three fundamental mechanisms in which plasmonic absorbers can absorb or trap EM radiation: through multiple scattering incidents from the metal NP/nanostructures, through the increased absorption cross-section generated by the tightly confined EM near-fields, and/or through surface polariton (SP)-guided modes [37]. Wang et al. demonstrated a novel selective solar coating as a solar–thermal absorber made of a tungsten, SiO_2_, and Si_3_N_4_ multilayer Fabry–Perot cavity structure [38]. It is still extremely challenging to achieve perfect absorption in the full solar spectral range (280–4000 nm) using a thin-film structure. Lui et al. proposed a forward step toward this challenge using thin-film refractory metal resonators (Figure 3a,b) [39]. Near-unity light trapping for the sun’s radiation was shown as the full-spectrum weighted solar absorption efficiency exceeded 98%. Excellent thermal emission with an average spectral efficiency of ~91% was achieved in the wavelength range of 280–4000 nm using a W-based absorber, suggesting this platform as a near-perfect blackbody-emitting source. The cooperative effect of the strong plasmonic resonances and the intrinsic broadband spectral responses of refractory metals such as Ti, V, and W resulted in excellent absorption efficiency. Moreover, all-dielectric PC-based high-Q-factor absorbers were proven to be potential candidates for designing near-unity light absorption within subwavelength-thin nanostructures, leading to the possibility of chip-scale thermophotovoltaic devices [40].

### 3.2. Plasmonic Nanostructure-based Solar Steam Generation

Owing to the limited freshwater supply (only 2.5% of all water on the earth) and the never-ending demand for freshwater associated with the increase in population, the development of efficient desalination techniques is urgently required. Solar energy is abundant, renewable, and eco-friendly; hence, solar-powered SG techniques are of great advantage among other desalination techniques such as pressure-driven ultrafiltration, electricity or mineral-combustion-based distills, membrane-based adsorption, or ion exchange. On the other hand, the extremely low efficiency of conventional SG techniques during bulk water heating is a major hindrance in solar energy utilization. An effective solution to overcome this limitation is to generate localized heat via confined electric fields caused by localized plasmonic resonance. The generated heat is focused to evaporate the water molecules positioned around the plasmonic NPs. The solar generation of vapor nanobubbles using plasmonic nanostructures was initially studied using single particles [42]. Subsequently, some types of free-standing assembled plasmonic films exhibited high efficiencies, owing to their plasmon coupling. A solar vapor generation device using a novel double-layer hydrogel was introduced by Sun et al. [41]. This device, named Ag-PSS-AG/AG (Figure 3c,d), was based on the hierarchical composition of Ag NPs and poly (sodium-p-styrenesulfonate) (PSS)-decorated agarose gel (AG). The device exhibited a synergetic effect between the two layers with high light-harvesting and water-transfer performances. As a result, an extremely high vapor generation rate of 2.10 kg m^−2^ h^−1^ and solar–thermal efficiency of 92.8% was achieved under 1 sun illumination. Inspired by the structure of a lotus leaf and flower, Chen et al. fabricated a porous 3D wooden flower decorated with Ag–polydopamine core-shell NPs, which exhibited a high vapor generation rate of 2.08 kg m^−2^ h^−1^ and an ultrahigh solar-to-vapor efficiency of 97.0% under 1 sun illumination [43].

### 3.3. Photonic Crystal-based Radiative Cooling

Manipulating the surface thermal emission by the thermal photonic design has received significant attention over the past decade. Passive radiative cooling methods are of remarkably higher interest because of their potential to decrease energy consumption by not requiring an external heat carrier such as fans or thermoelectric devices. Radiative cooling refers to the physical process by which thermal radiation is used to dissipate heat to a lower temperature body. A passive radiative cooling scheme reflects all the incident sunlight while simultaneously emitting the required thermal radiation in the midinfrared range without requiring any external active devices, such as fans or air conditioners. Recent works have shown that multilayer thin films can be manipulated to emit thermal radiation in the infrared transparency windows of the atmosphere while reflecting visible light, and as a result, daytime radiative cooling can be achieved under direct sunlight. Yao et al. proposed a PC-based reflection-type cooler composed of SiO_2_/Si_3_N_4_ nanoscale stacks, which not only compensates for the deficiency of metal reflectors by utilizing the bandgaps of the PCs but also enables a broadband emissivity peak in the atmospheric window [44]. By simultaneously engineering the forbidden band and thermal emission of PCs, they achieved near-perfect solar reflection and considerably high thermal emission at selective wavelength ranges. In another study, Kim et al. reported daytime radiative coolers with preserved structural colors using silica opals. Daytime radiative cooling up to 15 °C while preserving the nonabsorbing colorization was achieved through this versatile and large-scale coating of colloidal suspensions [45].

## 4. Photocatalysis

Increasing academic and industry attention has been given to photocatalysis as a direct process to harvest sunlight energy and convert it into chemical energy. Two main strategies to enhance photocatalytic activity are considered, including structural and compositional characteristic modifications. Semiconductor photocatalysts such as titania (TiO_2_) have band edges that meet the thermodynamic requirements for water-splitting reactions; however, its large optical bandgap leads to poor sunlight-harvesting efficiency. BiVO_4_ and Fe_2_O_3_ as narrow-bandgap semiconductors could not photogenerate sufficient thermodynamic potentials for water splitting. Various techniques to improve light utilization with wide-bandgap semiconducting photocatalysts have been investigated, including band potential shifting by doping and sensitization/incorporation with plasmonic metallic NPs, quantum dots, or PCs.

### 4.1. Plasmonic Photocatalysts

**Figure 4:**
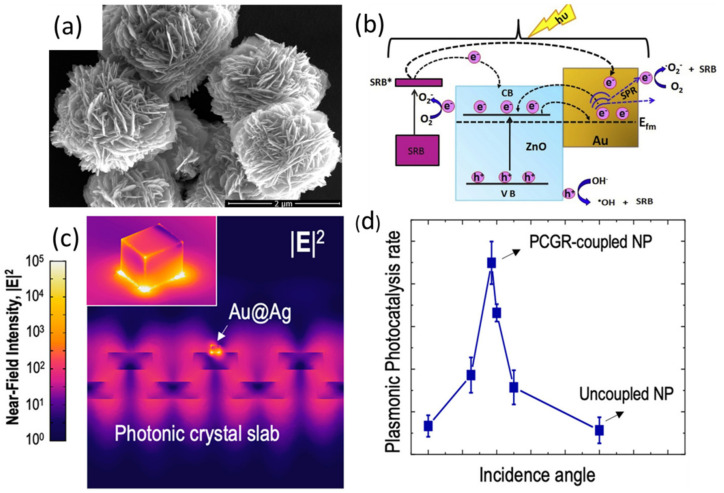
Hybrid plasmonic–photonic nanostructures. (**a**) FE-SEM images of ZnO/Au (1 mol %) nanoflower structure and (**b**) the transfer of the electron–hole pair in the ZnO/Au nanostructures shown in the schematic band diagram, responsible for the solar-irradiation-induced degradation of sulforhodamine B. Reproduced with permission from [50]. Copyright Elsevier, 2019. (**c**) Simulated near-field intensity of the Au@Ag–PC hybrid at θ = 3.5°, and (**d**) measured (1-reflectance-transmittance) spectra of the Au@Ag–PC hybrid at various incidence angles. Reproduced with permission from [53]. Copyright American Chemical Society, 2020.

Wide-bandgap semiconductors modified with noble metal (NM) exhibit the LSPR effect and have improved photocatalyst activity under visible-light irradiation. These materials, known as plasmonic photocatalysts, have been extensively investigated over the last few years [46,47,48,49]. Three main possible mechanisms of plasmonic-enhanced photocatalysis have been considered: (1) charge transfer; (2) energy transfer; and (3) plasmonic-heating-induced thermal activation. In the charge transfer mechanism, NMs act as plasmonic photosensitizers. LSPR excitation from the NM NPs assists the absorption of incident solar photons, and electrons are transferred from NMs to the conducting band of the semiconductors. Oxygen absorbed on the surface of the photocatalyst is reduced by these conducting band electrons, which is typical for aerobic photocatalysis for this type of conductor. On the other side, electron-deficient NM NPs need to replenish the zero-valent state by oxidizing organic compounds. Various studies have proven the possibility of this mechanism. Raji et al. investigated the solar-induced photocatalytic degradation of sulforhodamine B in the presence of plasmonic ZnO/Au nanostructures as a catalyst, finding that rapid degradation of the dye was achieved at the solar irradiation wavelength near 550 nm due to the incorporation of ZnO/Au plasmonic bands [50]. The high electronegativity of atomic gold resulted in good electron scavenging efficiency, enhanced light absorption with plasmonic effects, and the formation of a Schottky barrier in the ZnO/Au interface are the main reasons accounting for the enhanced photocatalytic activity of this structure (Figure 4a,b). The second mechanism, energy transfer between the two materials, could occur when their energy levels matched closely, even when they are separated by a dielectric interlayer. Plasmon resonance energy transfer (PRET) has been suggested for Au NPs deposited on TiO_2_, in which the SiO_2_ interlayer cannot inhibit the generation of electron–hole pairs in TiO_2_ [51]. The enhanced activity of these plasmonic photocatalysts is not a one-sided effect and depends on the characteristics of both the NMs, the semiconductor, and the cross-platform interactions between the two materials. The selective deposition with noble metals on specified surfaces or facets could improve the photocatalytic activity [52].

### 4.2. Photonic Crystal-based Photocatalysts

As an alternative, PCs possessing a photonic bandgap (PBG) and the effects of a slow photon can be optimized for efficiently utilizing incident photons in photocatalysts. At the edges of the PBG where scattering and light reflection occur, the formation of “slow photons” resulted in the reduction of the group velocity of photons. The photocarrier generation efficiency can be greatly enhanced by the localized “slow photons”. For example, slow photons localized in the titania skeleton of TiO_2_ photonic films can accelerate dye photodegradation kinetics via the spectral overlap between the dye electronic absorption and the low-energy edge of the inverse opal stopband, under the illumination of both the UV and visible range [54]. The TiO_2_ inverse opal films were also functionalized by graphene oxide nanocolloids (nanoGO), which further enhanced the catalytic effectivity by the dual incorporation of interfacial electron transfer from the TiO_2_ skeleton to the GO sheet materials.

Various bioinspired structures have been demonstrated to improve the efficiency of photocatalysts. Generally, synergizing structures such as natural porous PCs into the photocatalyst can increase the surface area and porosity for reacting and harvesting photons, respectively. Using a biotemplate based on a green leaf, the morph-TiO_2_ exhibiting significantly improved photocatalytic CO_2_ conversion was obtained [55]. Moreover, bioinspired structures such as the black scales on the butterfly wings showed enhanced light-harvesting and antireflection ability, which could be artificially fabricated as solar energy conversion systems. Rodríguez et al. proposed a biotemplating approach for precisely replicating Morpho nanostructures by the nanocrystalline deposition of ZnO coatings on the butterfly wings through low-temperature atomic layer deposition [56]. Optimizations of the coating thickness are required to fully utilize the photocatalytic activity of this structurally colored photocatalyst as a result of the balance between light absorption and photocatalytic quantum yield as the thickness of the coating layer is increased.

### 4.3. Hybrid Plasmonic–Photonic Photocatalysts

The combined application of PCs and plasmonic materials in microstructural materials can impact the photocatalytic processes in many ways. Firstly, surface plasmon resonance (SPR) enhancement by photonic Bragg resonance can increase the separation efficiency of the photogenerated pairs of electrons and holes. The interfacial potential of the heterojunction semiconductor can act as an electric field barrier accelerating the photogenerated charge carriers, increasing the interfacial electron transfer rates. Lastly, microstructural designs and plasmonic effects expand the light-harvesting range and improve the photocatalytic quantum efficiency. Various plasmonic PCs have been introduced for photocatalysis applications, ranging from water splitting [57] and the mineralization of organic pollutants [58]. A strategy toward the substantial enhancement of hot-electron generation at tunable narrow-band wavelengths using the hybridization of plasmonic and photonic resonance was introduced by Huang et al. [53]. By coupling the plasmon resonance of Ag NPs to the guided-mode resonance in a dielectric PC slab, the reduction conversion driven by hot electrons was significantly accelerated at a low illumination intensity (Figure 4c,d). A 3D-ordered thin-shell Au/TiO_2_ hollow nanosphere assembly from SiO_2_ nanospheres and titanate was demonstrated by Dinh et al. [59]. The fabricated Au/TiO_2_ hollow nanospheres showed an elevated surface area as well as enhanced light-scattering and photonic effects, resulting in significantly efficient visible-light absorption. Significantly enhanced visible-light-driven photocatalytic activities were observed in the designed photocatalysts compared to the conventional Au/TiO_2_ nanopowders.

## 5. Biosensors

Infectious diseases have always persisted as a source of burden in the global healthcare landscape. In particular, the outbreak of the novel coronavirus disease (SARS-CoV-2) has spread at a tremendous rate and poses a threat to public healthcare in almost every country worldwide. Rapid and reliable diagnosis of the disease has been one of the vital priorities for declining its spread as well as focusing on treatment for those infected. Currently, COVID-19 testing relies heavily on reverse transcription–polymerase chain reaction (RT-PCR) technology. Even though RT-PCR is considered the most sensitive and versatile method for detecting viral RNA, such as those from SARS-CoV-2 viral particles, it shows certain limitations and puts a strain on the healthcare infrastructure. Dependance on RT-PCR could overstretch the reagent supplies as trained personnel resources and lengthy processing times could lead to the delay of test results. For these reasons, biosensors are highly promising for providing reliable alternatives for real-time detection and continuous monitoring for disease biomarkers as well as other vital and environmental parameters. Among the current biosensing techniques, photonic- and plasmonic-based biosensors are applicable to various classes of analyte or clinical interest. PCs have distinct reflection wavelengths that are governed by the distance between the layers, spheres, or surrounding dielectric medium, which causes their specific color. For instance, if the periodicity of the crystal or the refractive index of the surrounding medium is changed by a biostimulus, the wavelength of maximum reflectance will also change. Hence, the effect provides a convenient aid for sensing, particularly if the effect can be made specific to the stimulus. SPRs are extremely sensitive to the local dielectric permittivity of the environment in contact with the metal surface. Therefore, significant shifts, even for few molecule-binding events of the stimulus, are often observed in the resonance spectrum of the plasmonic nanostructures. Such effects could lead to noticeable visual readout even by inexperienced operators. Moreover, the raw material cost and storage requirements of these biosensor-based methods could be further decreased. Some of these features make applications of photonic and plasmonic materials as biosensors much more realistic than other stimuli-responsive materials.

### 5.1. Photonic Biosensors

**Figure 5:**
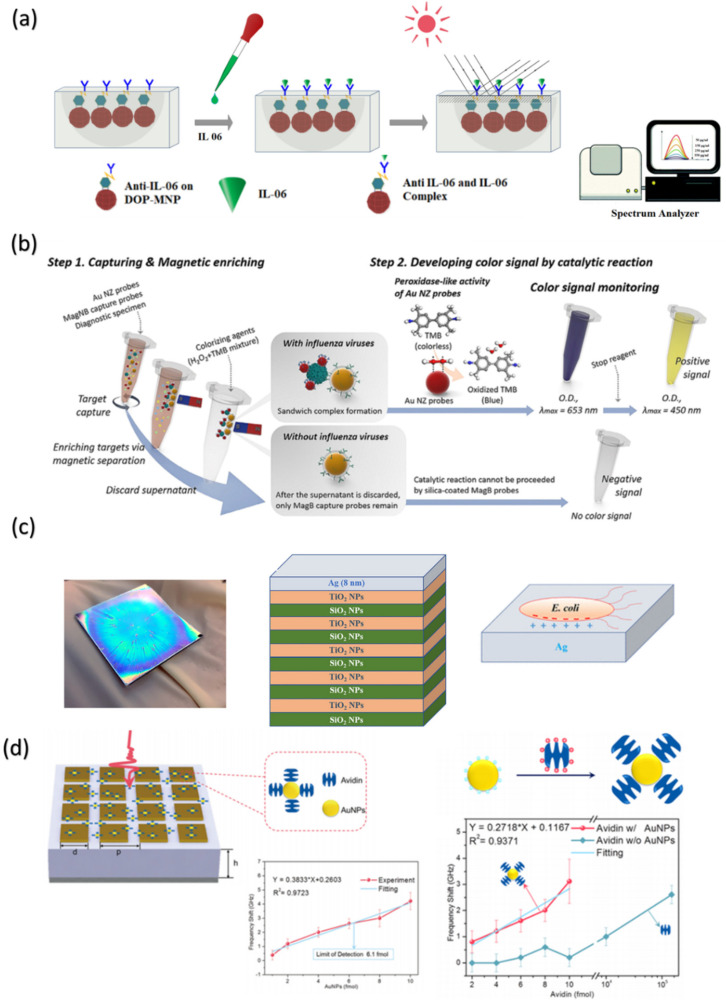
Biosensing devices based on photonic, plasmonic, and plasmon–photonic platforms. (**a**) Magneto-PC-embedded hydrogel detecting Interleukin-6 for real-time management of sepsis. Reproduced with permission from [61]. Copyright Royal Society of Chemistry, 2020. (**b**) Magnetic nanobead-based enzyme-linked immunoassay for the detection of influenza A virus. Reproduced with permission from [64]. Copyright American Chemical Society, 2018. (**c**) Hybrid plasmonic–photonic Bragg stacks with an 8 nm Ag capping layer for optical detection of the bacterial contaminants. Reproduced with permission from [65]. Copyright American Chemical Society, 2019. (**d**) Au NPs affect the peak shifting by conjugated avidin-Au NPs on the THz metamaterials. Reproduced with permission from [66]. Copyright American Chemical Society, 2016.

As mentioned earlier, conventional analysis methods for the detection and quantification of biomolecules, such as protein and genetic materials (DNAs and RNAs), have been well established over the past decades. Despite boasting unrivaled robustness and sensitivity for a wide range of applications and purposes, there is always a need for low-cost, disposable, and easy-to-use analysis tools such as test kits and colorimetric assays, which are not only tailored for clinical use but also practical research. PCs have emerged as a promising candidate for developing biosensors because of their ability to generate easy readout signals based on the changes in the diffraction wavelength and structural color in response to analyte stimulus signals. Generally, a PC-based biosensor composed of biological detecting components is either established on the surface or embedded in the PC structure. The responses of this type of device are mostly based on the changes in structures in the presence of the analyte target, which results in changes in the distances between the PC particles or the refractive index of the PC film [60]. For instance, antibody-detecting molecules were attached to the surface of a hydrogel matrix embedded with cobalt ferrite (CoF) and magnetite nanospheres, and this platform was deployed as a biosensor for real-time monitoring of interleukin-6. The hydrogel film produced a blue shift in the resonance wavelength when the antibody interacted with interleukin-6 at subpicomol levels [61] (Figure 5a). PC NPs can also be employed in more sophisticated devices in which PCs act as either responders or signal enhancers. Magnetic NPs with human chorionic gonadotropin (hCG)-conjugated antibodies were coadhered on the surface of an optical fiber interferometer, which enhanced the sensitivity of the microfiber sensor for the detection of hCG up to the highest-reported detection limit of 0.0001 mIU/mL [62]. A biointerface with the capability of preventing nonspecific adhesion could prevent false-positive or altered quantification caused by surface adherence of bacteria and biofilms. Bimodal interferometric waveguides (BiMWs) sensors with a silane–PEG–COOH surface-repelling bacterial adsorption showed selective label-free detection of *Pseudomonas aeruginosa* and *Staphylococcus aureus* as a proof of concept for the direct and rapid sensing of bacteria without sample pretreatment or signal amplification [63]. This type of sensor can be used in cases where infection detection and bacterial identification are crucial to ensure hygiene and safety, such as cleanrooms or emergency units, for patients and related personnel.

### 5.2. Plasmonic Biosensors

Plasmonic-based biosensors have recently been recognized as promising candidates for the development of next-generation diagnostic tools. These biosensors can be built based on various plasmonic modalities such as SPR, localized LSPR, plasmonic colorimetric assays, and SERS [67]. Advances in biosensor technology and design have shown that limitations in the flexibility and sensitivity of POC testing methods can be addressed through the involvement of plasmonic nanomaterials. Magnetic nanobeads with Au nanozyme probes capable of recognition, separation, and visualization of influenza virus A (Figure 5b) were developed as an immunosorbent assay for a simple but sensitive in situ POC diagnosis, as previously described by Oh et al. [64]. Two-dimensional (2D) Au nanoislands functionalized with complementary DNA receptors from SARS-nCoV-2 exhibit a dual-functional thermoplasmonic and LSPR sensing capability, which facilitates highly sensitive and accurate detection of SARS-nCoV-2 viral sequences [68]. An effective method to enhance the LSPR shift and increase the selectivity of the plasmonic biosensors is to increase the effective change in refractive index per molecular binding event. By controlling the pH level during the synthesis reaction, Au NPs were effectively implemented on a negatively charged cellulose strip surface [69] for classifying human cerebrospinal fluids from patients with cerebral vasospasm and hydrocephalus complications. Wang et al. developed a colorimetric sensing platform based on the etching of Au nanobipyramids (AuNBPs) using a horseradish peroxidase-encapsulated liposome-embedded magnetic bead probe to visually detect telomerase activity in HeLa cells. Limits of detection as low as 20 HeLa cells with the naked eye and 1 HeLa cell with LSPR measurement were achieved [70]. Fano-like resonances produced by the third-order LSPR mode were observed in the diffraction profile of an Au nanostripes grating [71]. With this effect, low-cost and large-area sensors were fabricated by soft lithography, which is applicable for sensing changes in refractive index at a resolution up to 10^−5^ refractive index units in a sensing area of 0.75 mm^2^.

For SERS-based sensing platforms, there is a crucial requirement for developing high sensitivity and reproducibility to improve surface uniformity. The SERS substrates fabricated using a facile spin-coating procedure at a low speed with a low volume fraction of ethanol resulted in a void-free hexagonal close-packed gold layer, which was deployed for Raman scattering analysis of human tears to detect breast cancer [72]. Highly uniform 3D plasmonic arrays with NP-spiked pillar structures were fabricated by vacuum deposition with selective nucleation and growth of spherical Au plasmonic NPs [73]. The modified nanopillar arrays were able to produce SERS signals twice as intense as the smooth nanopillars with the same thickness, which is applicable in plasmon-enhanced sensing diagnostics for sepsis and influenza virus.

### 5.3. Plasmon–Photonic Biosensors

Most studies on SPR-based sensing of biological and chemical analytes employ the Kretschmann–Raether prism. However, owing to their bulky and complicated design, which limits their application scenarios, plasmonic optical fibers, such as hollow-core photonic crystal fiber (PCF) sensors, are being increasingly used in SPR sensor development [74]. Hossain et al. developed a quasi-D-shaped external Au-coated PCF with significant amplitude sensitivity toward biological samples in the wavelength range of 1080–1560 nm [75]. Label-free PCFs based on Ag/TiN and Ag/ZrN configurations exhibit high sensitivity and linearity for quasitransverse magnetic and electric modes, which can detect unknown analytes as biological sensing devices [76]. Wang et al. introduced a large surface-to-volume ratio (SPR-PCF) with a silver-graphene layer lining, which is highly sensitive for refractive index dynamic detection ranges of 1.39–1.46 compared to previously described PCFs [77]. Overall, the optical spectra of PCF-SPR biosensors can be optimized by varying the structural parameters and core materials, which indicates their promising applications in biological and biochemical detection.

In addition to PCF-based applications, another interesting approach to plasmon–photonic biosensing is to employ nanostructures that consist of multiple plasmonic layers. Further, 2D and 3D nanostructures with point-defect cavities fabricated using nanoimprint lithography [78] were able to produce LSPR peak shifts in the presence of exosomes by confining and enhancing the EM field intensity through the hybrid coupling of plasmonic and PC modes. Bragg stacks fabricated by consecutive layers of SiO_2_/TiO_2_ NPs with a thin, 8 nm silver layer deposition (Figure 5c) showed selective interaction with bacterial cells, particularly Gram-negative bacteria such as *Escherichia coli*. This device, however, is only able to produce a 10 nm photonic structural shift in the presence of analyte cells, which was insufficient for visual colorimetric detection of *Escherichia coli* in practical uses [65]. Furthermore, while the LSPRs of plasmonic Au are mainly observed in the visible range, titanium (IV) carbide (Ti_3_C_2_) nanoparticles could produce LSPR that are tunable to near-infrared wavelengths [79,80]. In these studies, a curved photonic nanojet, dubbed “photonic hook,” was generated using a high-intensity narrow light beam generated by dielectric structures such as gold or Ti_3_C_2_ MXene nanoparticles. Compared to gold, photonic hooks generated from Ti_3_C_2_ nanopaticles showing high utilization of LSPR at a near-infrared wavelength range, which could facilitate noninvasive penetrable optomechanical sensing for biomedical testing applications.

### 5.4. Terahertz Biosensing and Imaging

Terahertz (THz) plasmonic structures with an operating bandwidth between that of transistors and lasers (0.1 THz < f < 10 THz), which lie between the microwave band and infrared band of frequencies, have received significant attention recently. THz spectroscopy has emerged as a promising approach for biosensing applications with many special features that are not often observed in other optical structures. THz plasmonic sensing enables high mobility, high biocompatibility, and less invasive analysis that can be designed as implants or wearable devices. The observation of vibrational modes of biological macromolecules (i.e., proteins, DNA, RNA) across the THz spectrum makes this bandwidth useful for sensing and monitoring purposes. The THz plasmonic metasurface commonly consisted of multiple recurring arrays of artificially engineered units, with electromagnetic properties beyond those of other natural materials. However, due to the THz plasmonic surface having a much smaller size than the THz radiation wavelength, nanoscopic molecules and microorganisms often show very low scattering cross-sections in the order of ~λ/100 [81].

Plasmonic nanoparticles (NPs), such as gold or silver, can function as metamaterial absorbers to improve sensitivity in the terahertz (THz) region. When plasmonic NPs are deposited on the THz plasmonic metamaterials, the localized field of the THz sensing device is improved, which increases the effectivity of the sensor in the THz regime [82]. Plasmonic NPs could also assist in the bonding between sensor surfaces and biomarkers as well as DNAs, RNAs, or proteins. When the Au NPs are coated with citrate, they strongly bind with positively charged biomolecules [83]. Xu et al. introduced Au NPs with plasmonic properties to improve the sensitivity of THz metamaterial structures by fabricating a simple form of a metamaterial consisting of square metallic patch arrays for THz spoof surface plasmon excitation. By altering the amount of Au NPs varying from 1 to 10 fmol compared to the uncoated substrate, linear shifts in THz peaks frequency related to the concentration of avidin were observed. Therefore, the introduction of plasmonic NPs has the potential to significantly enhance the detection sensitivity of THz metamaterials [66] (Figure 5d).

## 6. Conclusions

Optical functional nanostructures are suitable for a wide range of applications in photoenergy conversion, photocatalysts, and biosensing. This review introduced the mechanism and principles, as well as recent progress in the development of photonic and plasmonic nanostructures for energy and sensor applications. Incorporation of antireflective structures and plasmonic effects into the nanostructuring of light-harvesting devices could improve the solar energy conversion of photovoltaic devices, while photonic and nanophotonic structures could be developed as efficient photothermal conversion methods by manipulating electromagnetic waves on micro- and nanoscales. Moreover, hybrid photonic/plasmonic-based structures show highly enhanced photocatalytic activity as well as sensing capability, which could address some of the existing issues in solar energy conversion and biosensing systems. Finally, advances in solar energy conversion and biosensors could become the key to clean energy, efficient diagnostic tools, and infectious disease management in the near and distant future.
